# Incidence and direct hospitalisation costs of hip fractures in Vilnius, capital of Lithuania, in 2010

**DOI:** 10.1186/1471-2458-12-495

**Published:** 2012-07-02

**Authors:** Marija Tamulaitiene, Vidmantas Alekna

**Affiliations:** 1 Faculty of Medicine, Vilnius University, Vilnius, Lithuania; 2 National Osteoporosis Center, Vilnius, Lithuania; 3 State Research Institute Centre for Innovative Medicine, Vilnius, Lithuania

**Keywords:** Direct costs, Hip fracture, Hospital costs, Incidence, Treatment type

## Abstract

**Background:**

Few epidemiological data on hip fractures were previously available in Lithuania. The aim of this study was to estimate the incidence and hospital costs of hip fractures in Vilnius in 2010.

**Methods:**

Data were collected from the medical charts of all patients admitted to hospitals in Vilnius (population, 548,835) due to new low-energy trauma hip fracture, during 2010. The estimated costs included ambulance transportation and continuous hospitalisation immediately after a fracture, which are covered by the Lithuanian healthcare system.

**Results:**

The incidence of new low-energy trauma hip fractures was 252 (308 women and 160 men) per 100,000 inhabitants of Vilnius aged 50-years or more. There was an exponential increase in the incidence with increasing age. The overall estimated cost of hip fractures in Vilnius was 1,114,292 EUR for the year 2010. The greatest part of the expenditure was accounted for by fractures in individuals aged 65-years and over. The mean cost per case was 2,526.74 EUR, and cost varied depending on the treatment type. Hip replacement did not affect the overall mean costs of hip fracture. The majority of costs were incurred for acute (53%) and long-term care (35%) hospital stays, while medical rehabilitation accounted for only 12% of the overall cost. The costs of hip fracture were somewhat lower than those found in other European countries.

**Conclusion:**

The data on incidence and costs of hip fractures will help to assess the importance of interventions to reduce the number of fractures and associated costs.

## Background

With the growing size of the elderly population, osteoporotic fractures and disabilities resulting from them have a major impact on health. Several studies have quantified the global burden of osteoporosis by analysing the number of fractures: hip fracture sufferers were estimated at 56 million worldwide, with a female-to-male ratio of 1.6:1 [1-3]. As identified by a systematic review, osteoporotic fractures comprise a significant disease burden to society, particularly in developed countries [4]. The greatest number of osteoporotic fractures occurred in Europe (34.8%). Hip fractures are arguably the most serious and contribute most to the healthcare burden of osteoporosis; thus, a reliable estimate of their present and likely future incidence among both sexes is important in calculating the costs and resources needed to manage this problem [5]. Incidence and cost of fractures have been estimated in many countries and show substantial variation between populations, indicating the need for continued data collection [6,7].

Very little data are available about the incidence and consequences of fractures in Lithuania [8-10]. Osteoporosis in Lithuania is still under-reported on hospital discharge forms as co-morbidity, even in elderly patients with hip fractures. As yet, information about the economic consequences of treating fractures that resulted from osteoporosis has not been published in Lithuania.

The purpose of our study was to estimate the incidence and the associated direct hospital costs of hip fractures in Vilnius, the capital of Lithuania, in 2010.

## Methods

### Description of the health insurance financing system in Lithuania

The healthcare system in Lithuania covers the whole population. In compliance with the Law on Health Insurance, the State Patients’ Fund at the Ministry of Health guarantees the provision of personal healthcare services, including medical aid, rehabilitation and nursing.

The hospital financing mechanism is used for acute inpatient care, rehabilitation and long-term hospital care. All patients with hip fracture who need hospitalisation are treated in orthopaedic departments at government hospitals. No patients with hip fractures were admitted to private hospitals as none of these hospitals had an orthopaedic department in 2010.

### Sample

A retrospective cohort study was conducted for Vilnius citizens over 50-years of age that had sustained a hip fracture from January 1st to December 31st, 2010. All individuals whose hip fracture occurred as a consequence of minor- to moderate-trauma were included in this study. Exclusion criteria were non-Vilnius citizenship, high-energy trauma, primary bone diseases and bone metastatic disease. Re-admissions for the same fracture were excluded when calculating the incidence.

For the purposes of this study, we included information on two Vilnius hospitals with orthopaedic departments where all cases of hip fracture are treated. Hip fractures were validated by radiographs and were defined by the following International Classification of Diseases-10 (ICD-10) codes: S72.0 – fracture of femoral neck; S72.1 – pertrochanteric fracture; and S72.2 – subtrochanteric fracture. For each hospital discharge, the information recorded included age, gender, mode of entry, diagnosis, fracture circumstances, type of surgery, length of stay and discharge place. A medical chart review was carried out by research personnel trained to extract the required data. The study protocol was approved by the local Ethical Committee.

### Population data and incidence

Gender- and age-specific incidence rates for hip fractures were calculated for the population of Vilnius city. Vilnius has a stable Caucasian population of 548,835 (2010) and its own health-service. Population data were obtained from the Department of Statistics of the Republic of Lithuania (Statistics Lithuania) as of 1st January 2010 [11]. The study population was subdivided by gender and into eight age-groups of five years. Furthermore, the data were also analysed for larger age-groups: 50–64 years; 65 years and over; and 80 years and over. The incidence data were expressed as the number of cases per 100,000 inhabitants.

### Costs estimation

We calculated direct hospitalisation costs as comprising ambulance transportation, acute hospital stay, inpatient and outpatient rehabilitation, and long-term hospital care. The costs were calculated based on the reference price list of the State Patients' Fund. The mean cost of transportation to the hospital by ambulance was provided by Vilnius Emergency Medical Station. All costs were computed at 2010 prices in local currency terms (LTL) and were converted to Euros (EUR) using the fixed exchange rate (3.4528 LTL/EUR).

### Acute hospital stay costs

Analysis of the hospitalisation cost for hip fractures was patient-specific according to the treatment type, since the cost in each case was dependent upon the type of treatment rather than the length of stay. We therefore show four types of hospitalisation costs: non-surgical treatment; internal fixation by plate; internal fixation by screw; and hip replacement. The costs for acute hospital stay included surgery, intensive care, implants used (plate, screw or hip prosthesis), medical staff, radiological and laboratory services, physiotherapy, medication, nursing care and meals.

### Estimation of direct hospital costs related to rehabilitation and long-term care

We have also provided an estimate of post-fracture rehabilitation costs and direct costs of stay at a long-term care hospital. The cost of rehabilitation was based on the *per diem* rate (different for inpatient and outpatient rehabilitation) combined with the patient’s length of stay. The analysis of direct costs was conducted assuming that they did not exceed the average values. A fixed money amount was applied to all cases if the long-term care was provided at a hospital following hip fracture. The costs for both kinds of hospital included the physician, rehabilitation procedures, nursing, facility services, meals and medication.

We have added all these costs to calculate the subtotal estimated cost of each type of treatment. The mean cost per patient was calculated as the subtotal cost divided by the number of patients. The overall costs were calculated as a sum of costs for initial hospitalisation and costs of re-admission for the same fracture during the same year. In the case of re-admission, all the costs derived from subsequent hospitalisations were added to the costs of the treatment type that was used during the initial hospitalisation.

Statistical analysis: means and percentages were used to describe the data. All data were analysed by Statistical Package for Social Science (SPSS version 18.0 for Windows).

## Results

### Number of hip fractures and incidence

In Vilnius, 667 hospitalisations for hip fractures occurred during 2010. Of these, 517 individuals were citizens of Vilnius aged 50-years or more; those that were admitted due to new hip fracture have been included in this study according to the inclusion and exclusion criteria (441 patients; 335 women and 106 men). The mean age of these patients was 78.2 ± 10.2 years. The number of hip fractures and incidences by age and sex are shown in Table 1.

**Table 1:** Number and incidence of low-trauma hip fractures by age in women and men in Vilnius, in 2010

**Age (years)**	**Population**		**Number of hip fractures**		**Incidence (per 100,000)**	
	**Women**	**Men**	**Women**	**Men**	**Women**	**Men**
50-54	21920	16716	4	4	18	24
55-59	19405	13678	13	8	67	58
60-64	17023	10268	20	9	117	88
65-69	14489	8810	17	11	117	125
70-74	13771	7405	36	17	261	230
75-79	10127	5158	49	17	484	330
80-84	7619	3074	84	19	1103	618
≥85	4396	1147	112	21	2548	1831
Total	108750	66256	335	106	308	160

The majority of fractures (87%) occurred in subjects older than 65 years, and the peak number of fractures occurred in individuals above 80-years of age. Seventy six per cent of the patients suffering hip fractures were women. The highest female/male ratio (5.3:1) was observed in subjects aged 85-years or more.

According to the population data provided by Statistics Lithuania for the 1st January of 2010, the population of individuals older than 50 was 175,006 in the city of Vilnius. Thus, the global incidence of hospitalisation due to new hip fractures was 252 (308 women and 160 men) per 100,000 inhabitants of Vilnius over 50-years of age. Fractures were 1.9-fold more frequent in women than men. There was an exponential increase in the total incidence of hip fractures with increasing age (Table 1). Hip fracture incidence (per 100,000 individuals) was 591 above the age of 65 and 1107 above the age of 75 in women, and 332 and 608 in men, respectively.

### Type of treatment and post-hospital medical care

Orthopaedic surgical treatment was the main type of hip fracture management, and only 12 subjects (3%) were treated non-surgically. The most frequent type of surgical procedure used was osteosynthesis: internal fixation by plate was performed in 110 women and 47 men (33% and 44%, respectively); and internal fixation by screw was performed in 162 women and 51 men (48% and 48%, respectively). Among 59 persons who underwent hip replacement, 54 were women and five were men. The results indicate that the overall in-hospital mortality for patients with hip fracture was 2%. Among those managed conservatively, the mortality was 25%, which significantly exceeded the in-hospital mortality after surgical treatment of hip fracture (1%).

After a stay in acute hospital beds, 145 patients (33%) went to another facility for inpatient rehabilitation and five patients underwent outpatient rehabilitation. Of all cases, 139 individuals (32%) went to the long-term care hospital and 145 (33%) were discharged home. The outcome of patients after acute hospital stay differed according to treatment type. In the cases of non-surgical management, all the patients who survived were discharged home or moved to long-term care hospital. About one-third of those who underwent internal fixation moved to rehabilitation facilities, whereas rehabilitation was performed in almost all cases of hip replacement. The distribution of subsequent hospitalisations varied by age and by gender. The proportion of men who underwent rehabilitation after the arthroplasty was higher than that of women (100% and 82%, respectively). Women were sent to a long-term care hospital more frequently than men, especially in the cases of internal fixation by screw. The exception was in two age-groups (75–79 years and over 85 years), where a larger proportion of men were discharged to a long-term care hospital. There were 12 cases of re-admission (almost 3%). Arthroplasty was performed on all re-admitted patients. Of these, ten individuals underwent inpatient rehabilitation, one woman was discharged home, and one woman moved to the long-term care hospital.

### Direct medical costs related to hospitalisations

The overall estimated cost of hospitalisations due to hip fractures in Vilnius was 1,114,292.05 EUR for the year 2010. A mean cost per case was 2,526.74 EUR. The total cost for acute hospital stay at the orthopaedic department, together with ambulance transportation, was 588,162.07 EUR and this accounted for 53% of the overall direct hospital costs for hip fractures. The mean total cost of acute hospital stay (including ambulance transportation to the hospital) was 1,333.70 EUR. Long-term care at special hospitals (396,255.09 EUR) was the second most important component of overall expenditure (35%), and this was more than three-times larger than the total rehabilitation cost (129,874.89 EUR). The mean total cost of long-term care was 898.54 EUR; the mean total cost of rehabilitation was 294.50 EUR.

We have also analysed the direct hospital costs for each type of treatment. The estimated costs of hip fractures by type of treatment during the acute period and the type of further measures taken are shown in Table 2. The costs of re-admission were assigned to the costs of initial type of treatment irrespective of further management.

**Table 2:** The estimated total costs of hip fractures depending on the type of treatment (in EUR)

**Medical services**	**Type of treatment used at the initial hospitalization**								**Total**	
	**Non-surgical**		**Internal fixation by plate**		**Internal fixation by screw**		**Arthroplasty**			
	**n**	**Cost**	**n**	**Cost**	**n**	**Cost**	**n**	**Cost**	**n**	**Cost**
**Initial hospitalization**										
Ambulance	12	718.06	154	9215.11	204	12207.03	58	3470.63	428	25610.83
Acute hospital stay	12	6779.67	157	169596.71	213	269611.01	59	96949.37	441	542936.76
In-patient rehabilitation	-	-	44	36529.38	54	44831.51	47	39020.02	145	120380.91
Out-patient rehabilitation	-	-	2	476.74	1	238,37	2	476.74	5	1191.85
Long term care hospital	4	11321.59	54	152841.52	80	226431.88	1	2830,05	139	393425.04
Subtotal costs	12	18819.32	157	368659.46	213	553319.8	59	142746.81	441	1083545.39
Mean subtotal cost	-	1568.28	-	2348.15	-	2597.75	-	2419.44	-	2457.02
**Readmission for the same fracture**										
Ambulance	1	59.84	-	-	-	-	-	-	1	59.84
Acute hospital stay	1	1622.89	-	-	11	17931.75	-	-	12	19554.64
In-patient rehabilitation	1	830.21	-	-	9	7471.92	-	-	10	8302.13
Long term care hospital	-	-	-	-	1	2830.05	-	-	1	2830.05
**Overall costs**	12	21332.26	157	368659.46	213	581553.52	59	142746.81	441	1114292.05
**Overall mean cost**	-	1777.69	-	2348.15	-	2730.30	-	2419.44	-	2526.74

The highest mean cost of acute hospital stay (with ambulance transportation added) was found in cases of arthroplasty, and this cost reached an average of 1,702.03 EUR. The mean costs of hospital stay for other treatment types were lower: 1,323.08 EUR for internal fixation by screw; 1,138.93 EUR for internal fixation by plate; and 624.81 EUR for non-surgical management. It was found that in cases of arthroplasty, the cost of acute hospital stay comprised 70% of overall expenditure; for internal fixation by screw, acute hospital stay accounted for 52%; in cases of internal fixation by plate, 49%; and in non-surgical treatment it was 43%.

In cases of arthroplasty the costs of rehabilitation in special facilities comprised 28% of overall costs, whereas in cases of internal fixation, rehabilitation costs comprised about 10% of the total, and, in non-surgical treatment, 4% of overall costs. Long-term care hospitals were most costly among patients treated conservatively (53% of overall costs), followed by cases of internal fixation by plate (41%) and internal fixation by screw (39%). In patients who underwent arthroplasty, the costs for long-term care accounted for 2% of overall costs. When the estimated mean costs were compared, the results showed that the internal fixation by screw was the most costly type of treatment. The difference became more evident when the costs of re-admission were added and the overall costs were estimated (Table 2).

Hospital costs of hip fracture have been analysed by age-groups and gender, and the data is shown in Table 3.

**Table 3:** Hospital costs (in EUR) induced by hip fractures, by age and gender

**Age (years)**	**Number of patients**		**Overall costs, including the cases of readmission**		**Mean cost**	
	**Women**	**Men**	**Women**	**Men**	**Women**	**Men**
50-54	4	4	11860.21	5746.96	2965.05	1436.74
55-59	13	8	24817.77	14138.97	1 909.06	1767.37
60-64	20	9	48863.39	14777.25	2443.17	1641.92
65-69	17	11	42251.05	25164.17	2485.36	2287.65
70-74	36	17	89229.97	38999.13	2478.61	2294.07
75-79	49	17	124142.90	45290.35	2533.53	2664.14
80-84	84	19	230090.95	48567.48	2739.18	2556.18
≥85	112	21	292722.32	57629.18	2613.59	2744.25
Overall	335	106	863978.56	250313.49	2579.04	2361.45

Over three-quarters (78%) of the overall costs of hip fractures resulted from incidences among women. When calculated by age group, the total costs generally increased with age. In women, the costs at the ages of 70–74 were more than twice the costs at the ages of 65–69, and then sharply increased with increasing age. Women above the age of 65 years accounted for 90% of women’s total costs, and men of the same age accounted for 86% of men’s total costs.

Finally, we estimated the total cost of hip fractures in men and women depending on treatment type (Table 4).

**Table 4:** Hospital costs (in EUR) induced by hip fractures, by treatment type

**Type of treatment**	**Number of patients**		**Overall costs**		**Mean cost**	
	**Women**	**Men**	**Women**	**Men**	**Women**	**Men**
Non-surgical	9	3	16547.52	4784.74	1838.61	1594.91
Internal fixation:						
by plate	110	47	258713.68	109945.78	2351.94	2339.27
by screw	162	51	458615.19	122938.33	2830.96	2410.56
Arthroplasty	54	5	130102.17	12644.64	2409.30	2528.93
Overall	335	106	863978.56	250313.49	2579.04	2361.45

Internal fixation by screw was the most costly treatment type in both women and in men (53% and 49% of overall costs, respectively). We also calculated the highest mean cost in women for this type of treatment. A different distribution of mean costs by treatment type was found in men, and the highest mean cost was calculated for arthroplasty; this cost was found to be higher in men than in women.

## Discussion

This study evaluated incidence of low-trauma hip fractures in Vilnius. This is the first attempt to estimate the direct costs related to the treatment of hip fractures in Lithuania. There were 441 new low-energy trauma hip fractures in individuals over 50-years of age in the city of Vilnius between January 1st and December 31^st^ 2010. As in previous epidemiological studies conducted in European countries, we also found that hip fractures in Vilnius were more numerous among women than men. The peak number of hip fractures occurred after the age of 80, which is similar to the data of other studies [4,12,13]. Our results show that the overall incidence of new low-energy trauma hip fractures during 2010 was 252 per 100,000 inhabitants of Vilnius aged over 50 years, and there was an exponential increase in hip fracture incidence with increasing age – a fact which has been widely described in medical literature.

The comparison of our data with data of other studies is challenging due to differences in study year, size of the study region or size of population investigated, different age groups and data presentation. Several previous studies were based on the analysis of health registries, hospital databases, nationwide health insurance databases and outpatient data, and so the results are not directly comparable with our data obtained by reviewing the medical records of every patient.

Although the literature review revealed relevant differences in study methods, the incidence of fractures is the most suitable index to compare different countries. Northern European countries have a higher hip fracture incidence than southern European countries [3,6], and different incidence rates were also seen among regions in the same country [12,14-16]. We have found the hip fracture incidence in Vilnius to be lower than in Portugal, France, Austria, Switzerland and Hungary [14,15,17-19], and especially lower than in Scandinavian countries [6,20-22]. Approximately the same results as ours were reported in studies from Greece [23] and Germany [16]. Lower incidence rates were reported from Spain [24] and Poland [25], and in another study comparing East and West Germany [26]. A similar pattern was seen when the overall incidence of hip fractures among women in Vilnius was compared with figures from equivalent studies in Europe. The overall female to male ratio of hip fractures was 1.9:1, which is similar to that in Poland [25], Hungary [19], Denmark [20] and Greece [23], but is lower than in Austria [17], Germany [16] and Norway [27]. This lower ratio may be explained by the higher incidence of hip fractures in men in Vilnius compared to other countries. The overall incidence of hip fractures (per 100,000 inhabitants) among men in Vilnius (160) is higher than in Germany (110.2) [26], Portugal (129.39) [14], Poland (89) [25] and Spain (100.4) [24], and the differences between our data and that from other countries for men are not as great as for women.

The geographic differences could be due to environmental and/or socioeconomic factors. It would be interesting to compare regions similar to Vilnius with respect to these factors, but we were not able to find any data on fracture incidence in neighbouring countries (i.e. Latvia, Estonia and Belorussia) in the literature. The only available study is from Poland in 2005, which reported one of the lowest incidence rates in Europe: 165/100,000 fractures for women above 50 years, and 89/100,000 for men [25]. Although these figures are lower than ours, the results are not directly comparable due to differences in the study population. The hip fracture incidence rates in Poland are based on national data whereas we analysed data only for the city of Vilnius. In 2006, Johnell and Kanis computed the incidence of hip fracture worldwide for men and women aged 50-years or more in five-year age intervals, from the year 1990 onwards [4]. Country-specific data was used for subregions within the same region of the global burden of osteoporosis. The only estimate available for the subregion in which Lithuania was included was from Hungary. The incidence of hip fractures in Vilnius, the capital of Lithuania, is lower than in Hungary, where the total incidence of hip fractures per 100,000 individuals was 343: 430 in women and 223 in men aged over 50 years, although the female/male ratio was approximately the same in both studies – 1.93:1. Thus, the results of our study fill a gap in the data from Europe and also the "blank areas" in the world map where there is a lack of data regarding the incidence of fractures [6].

To the best of our knowledge, this study is the first attempt to estimate the global burden of hip fractures in Lithuania, whereas several studies estimating medical expenditures for hip fractures or other osteoporotic fractures have been conducted during the past two decades [13,15,18,28-33]. The results of our study show that the overall direct hospital costs in Vilnius were as high as 1,114,292.05 EUR in 2010. These costs included the costs of ambulance transportation and continuous hospitalisations immediately after a hip fracture, which are covered by the Lithuanian healthcare system. Analysis of our data shows that the majority of costs were incurred for acute hospital stay (53%) and stays at a long-term care hospital (35%) versus 12% for medical rehabilitation.

We calculated the mean overall hospital cost for treating a hip fracture to be 2,526.74 EUR. This cost is low compared to many other countries. The costs of inpatient acute hospital care for hip fracture varies widely and was estimated at 5,983 US dollars in Turkey [34], 4,365.50 US dollars per case in Mexico [32], 14,616 CHF per case of hospitalisation for any osteoporotic fracture in Switzerland [18] and from 8,048 to 8,727 EUR in France [15]. As a study from Belgium reported, the mean cost of acute hospital stay was 8,667 EUR and the mean one-year, hip-fracture-related post-hospitalisation extra costs were 6,636 EUR [29]. In Sweden, the mean fracture-related cost the year after fracture was estimated at 14,221 EUR [33]. The mean total cost of hip fracture per patient per year in the United States was estimated at 26,856 US dollars [30], and in Canada the mean one-year cost was estimated at 26,527 Canadian dollars [31]. It is difficult to compare our results of cost estimates with the results of studies in other countries. Differences in the costs of hip fractures between countries may be attributed to differences in the economic development level, the healthcare financing system, price levels, rehabilitation rates and the length of hospital stay. Moreover, studies have used different methodology and data collection procedures. Some studies report only the direct hospital costs; others include outpatient costs, the costs associated with rehabilitation and residency at nursing homes, or all fracture-related costs in the following year. It would be reasonable to compare the incidence and cost of hip fractures in neighbouring countries. However, to our knowledge, no studies focusing on the economic burden of hip fractures in these countries have yet been published.

In Lithuania, acute care and long-term care hospitals, as well as rehabilitation units, are public institutions, and the costs of items do not vary from institution to institution, but only from treatment to treatment. We have estimated the direct hospital costs across age groups, gender and treatment types. The results show that the greatest part of the burden of hip fractures was incurred by women, and that the costs increased with age among both genders. The greatest part of the expenditure (90% in women and 86% in men) was accounted for by people above 65-years of age, and costs for individuals aged 85 and older account for almost one-third of all direct hospital costs. Similar percentages were reported in other studies [13,18,30,32,33]. When the costs of four types of treatment provided for hip fractures were analysed, our data showed that the highest overall expenditures were for internal fixation by screw, followed by internal fixation by plate. The major factor affecting the overall expenditures was the cost of stay in a long-term care hospital. In both types of treatment, long-term care constituted a large component of overall costs (39% in fixation by screw and 41% in cases of fixation by plate), whereas long-term care accounted for only 2% of the overall cost among patients treated by arthroplasty. Another important component of the costs of internal fixation by screw was the cost of re-admission. All 12 individuals re-admitted for the same fracture underwent the arthroplasty. Moreover, ten of them were later discharged to a rehabilitation facility.

When the overall hospital costs were considered, the estimated cost of arthroplasty was low since only 13% underwent this type of treatment. Although it is evident that acute hospital stay in the case of hip replacement is the most expensive when comparing the estimated costs of the treatment types, arthroplasty did not increase the mean overall hospital costs associated with hip fracture. The mean cost (2,419.44 EUR) was lower than the cost of treatment by internal fixation by screw (2,730.30 EUR).

The primary strength of this analysis is that all the hip fractures that were managed in the hospitals in Vilnius in 2010 were counted and analysed in our study. In Lithuania, persons suffering from hip fracture almost always receive hospital care, and it is difficult to model the situation if a person could evade medical aid and admission to an orthopaedic department. No patients with hip fracture were admitted to any of the private hospitals. While it is possible that a few inhabitants of Vilnius with hip fractures could be treated outside the city, this would not significantly influence the results of our study. It is more common for people of the surrounding regions to come to Vilnius for healthcare than *vice versa*.

Another major strength of the present study is the accuracy of information provided. All medical records - i.e. not discharge data - were reviewed by specially trained staff using the same data collection form and eliminating the possibility of counting fractures twice. All cases of re-admission for the same fracture were excluded from fracture incidence evaluation but added when fracture costs were calculated.

A limitation of our study is that the impact of other direct costs related to hip fractures was not estimated. The costs we have calculated are restricted to hospital costs and do not include direct outpatient medical costs, reimbursement for drug prescriptions and assistive devices. To ensure a more realistic estimation, costs of acute non-orthopaedic complications such as pneumonia, deep vein thrombosis, infection and co-morbidity should also be included in the calculation. Some patients could be hospitalised in a long-term care hospital or rehabilitation centre not immediately after the acute hospital stay, but later. Also a few cases of re-admission for the same fracture might not have been recorded if the individual was hospitalised at another, non-orthopaedic department or another hospital. The aim of this study was to estimate direct hospital and post-hospital medical costs only. The overall (direct and indirect) medical costs of hip fractures exceed those calculated in this study and are as yet unknown to us.

## Conclusions

This study contains useful information about the incidence and costs of hip fractures in Lithuania. This paper provides an estimate of hospital costs covered by the government and highlights the differences in the costs of different treatment types. This information on the current costs of fractures will aid clinicians, policy makers, and healthcare organisers in Lithuania to assess the importance of interventions to reduce the associated costs. Also, the data of this study have made possible to compare major healthcare problems in Lithuania. As the socio-economic burden of osteoporosis and related fragility fractures is expected to increase remarkably during the next two decades due to the ageing of the general population, it is necessary to identify resource needs.

## Competing interests

The authors declare that they have no competing interests.

## Authors’ contributions

MT and VA contributed equally to study design, data analysis, and writing of manuscript. Both authors read and approved the final manuscript.

## Pre-publication history

The pre-publication history for this paper can be accessed here:

http://www.biomedcentral.com/1471-2458/12/495/prepub
